# Transfer of functional microRNAs between glioblastoma and microvascular endothelial cells through gap junctions

**DOI:** 10.18632/oncotarget.12136

**Published:** 2016-09-20

**Authors:** Dominique Thuringer, Jonathan Boucher, Gaetan Jego, Nicolas Pernet, Laurent Cronier, Arlette Hammann, Eric Solary, Carmen Garrido

**Affiliations:** ^1^ INSERM, U866, Faculty of Medecine, 21000 Dijon, France; ^2^ CNRS ERL 7368, STIM laboratory, 86022 Poitiers, France; ^3^ University of Bourgogne-Franche-Comté, 21000 Dijon, France; ^4^ INSERM, U1170, Institut Gustave Roussy, 94508 Villejuif, France; ^5^ CGFL, 21000 Dijon, France

**Keywords:** gap junction, connexin, microRNA, tubulogenesis, glioblastoma

## Abstract

Extensive invasion and angiogenesis are hallmark features of malignant glioblastomas. Here, we co-cultured U87 human glioblastoma cells and human microvascular endothelial cells (HMEC) to demonstrate the exchange of microRNAs that initially involve the formation of gap junction communications between the two cell types. The functional inhibition of gap junctions by carbenoxolone blocks the transfer of the anti-tumor miR-145-5p from HMEC to U87, and the transfer of the pro-invasive miR-5096 from U87 to HMEC. These two microRNAs exert opposite effects on angiogenesis *in vitro*. MiR-5096 was observed to promote HMEC tubulogenesis, initially by increasing Cx43 expression and the formation of heterocellular gap junctions, and secondarily through a gap-junction independent pathway. Our results highlight the importance of microRNA exchanges between tumor and endothelial cells that in part involves the formation of functional gap junctions between the two cell types.

## INTRODUCTION

Among all brain cancers arising from transformed glial cells, grade IV glioblastoma (GBM), as defined by the World Health Organization, is the most prevalent and aggressive [1]. The diffusely invasive nature of these tumors precludes their complete surgical resection, which inevitably leads to tumor recurrence and patient death [2]. Glioblastoma cells migrate onto normal brain microvessels for invasion and tumor growth [3]. They communicate directly with surrounding normal cells such as astrocytes, glia and endothelial cells, through the formation of gap junctions. These cell-to-cell interactions modify astrocyte phenotype [4–6] and promote tumor angiogenesis and tumor growth [7]. Therefore, gap junctions and their signaling are proposed as potential therapeutic targets in these patients.

Gap junctions are specific cell-to-cell channels formed by membrane proteins called connexins (Cx). Connexin43 (Cx43) is the major connexin expressed in human microvascular endothelial cells (HMEC), astrocytes and glioblastoma cells. Cx43 is specifically upregulated in the reactive astrocytes surrounding glioblastoma [8], suggesting that gap junctions at the tumor margins are involved in tumor cell invasion [4, 6, 8, 9] and tumor growth [10]. The precise role of gap junctions remains poorly understood. Nevertheless, microRNAs (miRs) were observed to be exchanged between glioblastoma cells [11] and normal astrocytes [4] through gap junctions. MiRs are small non-coding RNAs that modulate gene expression by affecting the translation of messenger RNAs (mRNAs) into proteins and inducing target mRNA decay [12–14]. A miR is single-stranded and ~21nucleotides long, forming a linear molecule with a diameter of ~1.0 nm, which is in the same order of the gap junction channel pore size [15, 16]. We have recently reported that miR-145-5p, which reduces glioma growth [17], could be exchanged between HMEC and colon cancer cells through gap junctions formed by Cx43 [18].

Here, we examined whether the formation of gap junctions could permit the exchange of specific miRs between glioblastoma and microvascular cells, and how this transfer could influence the endothelial cell function *in vitro* (i.e., leading to angiogenesis). We used the U87 human glioblastoma cell line and HMEC, and focused on two human mature miRs, namely miR-145-5p which is expressed in HMEC but not in U87 [17], and miR-5096 which is expressed in U87 [4] but not reported in HMEC. We demonstrate an exchange of these two miRs between the two cell types through the same gap junction pathway. The effects of miR-5096, whose transfer is transiently mediated by gap junctions, starts at earliest stages of cell-cell contact and may be amplified by signal spread among HMEC. Our results reveal that glioblastoma cells modify the behavior of microvascular cells through miR transfer.

## RESULTS

### Expression levels of miR-145 and miR-5096 in glioblastoma and microvascular endothelial cells

**Figure 1:**
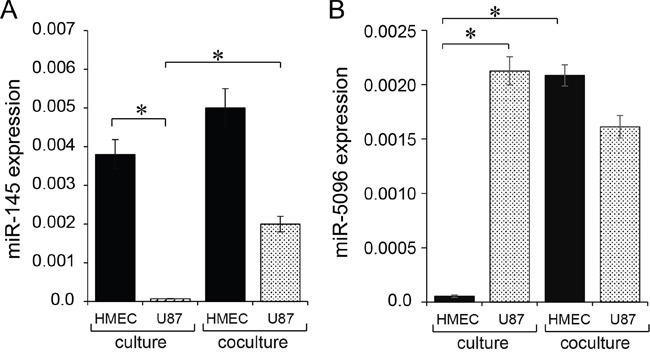
Expression of mature miR-145-5p and miR-5096 in microvascular endothelial cells (HMEC) and glioblastoma cell line (U87) **A, B.** Expression level of miR-145-5p and miR-5096 was measured by qPCR in HMEC (black) and U87 (dashed), cultured separately (left) or co-cultured (right) for 12 h (means ± SD; *P<0.05; *n* = 3). Levels of miR-145 or −5096 were measured relative to levels of U6 snRNA, as an internal control. For co-cultures, U87 were labelled with the fluorescent dye DiL-C18 (red cells), plated with unlabeled HMEC at a 1:1 ratio, and sorted by flow cytometry before analysis.

We first explored the basal expression level of miR-145-5p and miR-5096 in HMEC and U87 cells, in homotypic cultures. We observed that miR-145-5p was almost exclusively expressed in HMEC and could not be detected at a significant level in U87 (Figure 1A). In contrast, miR-5096 was mainly expressed in U87 and poorly detected in HMEC (Figure 1B). We subsequently labelled U87 with the cell tracker DiL-C18 [19], and co-cultured these cells with HMEC before flow cytometry sorting. As an additional control, the expression levels of these miRs were measured in U87 and HMEC, sorted immediately after being mixed, and were similar to that measured in cells cultured separately, i.e. miR-145-5p and miR-5096 remained poorly detected in U87 and HMEC, respectively (not shown). After 12 hours of co-culture, mir145-5p expression level was increased in both U87 (by 40%) and HMEC (by 20%) (Figure 1A). At the same time, miR-5096 expression level was significantly increased in HMEC while decreased by 20% in U87 (Figure 1B). These observations led us to explore miR exchanges between these two cell types.

### Micro-RNA exchange between endothelial and glioblastoma cells

**Figure 2:**
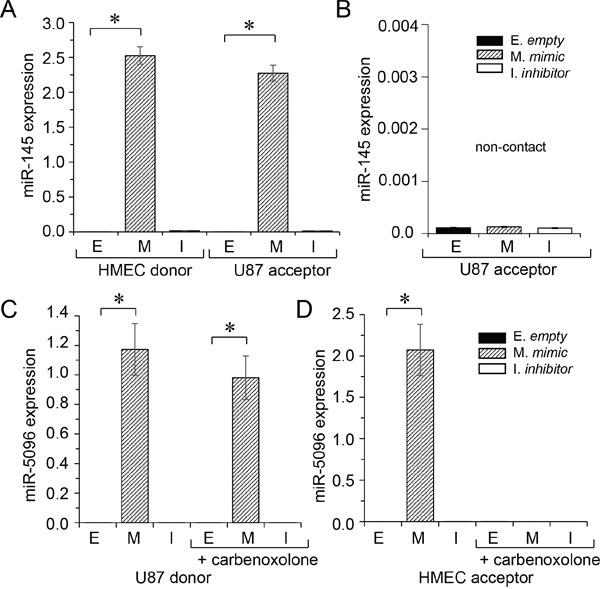
miRs transfer between HMEC and U87 Donor cells were loaded or not (E) with miR-mimic (M; 30 nM, hatched) or miR-inhibitor (I; 30 nM, white). The miR levels were determined by qPCR in donors (left) and acceptors (right), after 12 h of co-culture, and measured relative to U6 snRNA. Values are means ± SD of triplicate measurements from three experiments; *P<0.05 *vs* empty (Mann-Whitney U test and Kruskal-Wallis test; *n* = 3). **A.** Transfer of miR-145 from HMEC to U87. **B.** Abolition of miR-145 transfer to U87 when cells are co-cultured in transwell plates (non-contact). **C.** Transfer of miR-5096 from U87 to HMEC. **D.** Inhibition of miR-5096 transfer to HMEC by carbenoxolone (100 μM), a gap junction blocker. Note that carbenoxolone does not affect miR-5096 level in transfected U87.

To determine whether miR-145-5p was transferred from endothelial to cancer cells, we transfected HMEC with a miR-145-5p mimic (30 nM) before culturing them with DiL-C18-labelled U87 (ratio 1:1) for 12 hours. The two cell types were subsequently sorted by flow cytometry and miR145-5p expression was measured in each population. Both HMEC and U87 expressed high levels of miR-145-5p (Figure 2A). To evaluate the contribution of cell-to-cell contact in this transfer, we cultured U87 with miR-145-5p-transfected HMEC in transwell plates to prevent any cell-cell contact. In these non-contact conditions, we failed to detect any increase in miR-145-5p expression level in U87 (Figure 2B). These results indicate that U87 do not ingest extracellular miR-145-5p, either free or incorporated into soluble exosomes [18].

We performed the same experiment by transfecting U87 with miR-5096 mimic (30 nM) before culturing them with DiL-C18-labelled HMEC (ratio 1:1) for 12 hours. High levels of miR-5096 were also detected both in U87 and HMEC (Figure 2C, 2D). No increase in miR-5096 expression level in HMEC was observed in non-contact co-cultures with U87 (not shown). To evaluate the contribution of gap junctions to miR-5096 transfer from glioblastoma cells to HMEC, co-culture was made in the presence of carbenoxolone, in order to block the gap junction intercellular communication (GJIC) [11, 20]. Clearly, inhibition of GJIC prevented the miR-5096 increase in HMEC (Figure 2D), *i.e.*- the miR-5096 expression in HMEC remained very low in the presence of carbenoxolone (0.761±0.4 × 10-4) as compared to cells cultured alone (0.801±0.4 × 10-4, n=3; P>0.5). Because Cx43 is mostly involved in GJIC between HMEC and U87 [7], we knocked down Cx43 expression in U87 by using specific siRNA (see supplementary Figure S3A). The down regulation of Cx43 in U87 prevented the transfer of miR-5096 to HMEC (see supplementary Figure S3C). It is to note that carbenoxolone also prevented the transfer of miR-145-5p from HMEC to U87; the miR-145 expression levels in U87 were 2.2742±0.43 and 2.21±0.1 × 10-4, respectively in the absence and in the presence of carbenoxolone (means ± SD; *P*<0.05; *n* = 3), *i.e.* a miR-145 level similar to homotypic U87 culture (2.601±0.1 × 10-4, n=3; P>0.5). Thus, the two miRs were exchanged through the same pathway.

### miR-5096 favors communication between glioblastoma and endothelial cells

**Figure 3:**
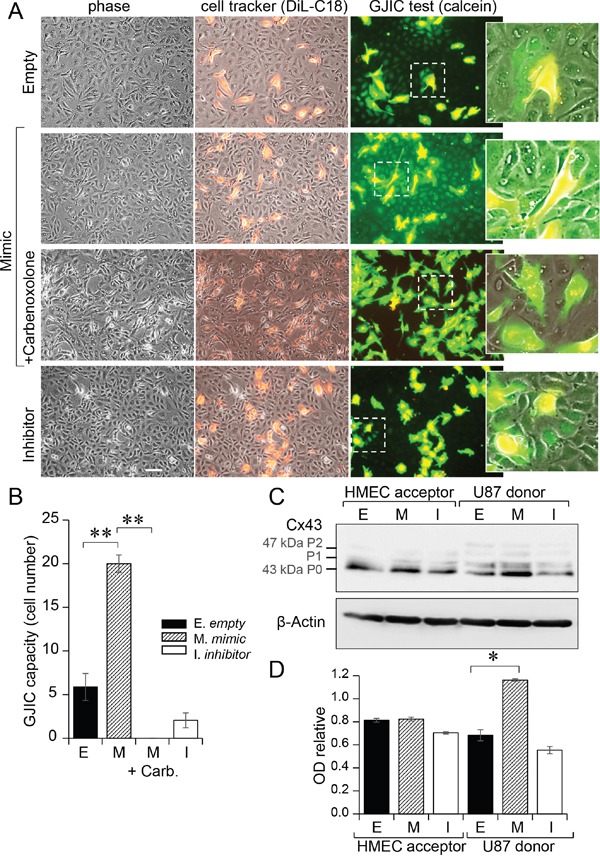
Gap junctions mediated miR-5096 transfer from U87 to HMEC **A.** Functional GJIC between U87 and HMEC. Donor U87, transfected or not (Empty) with miR-5096-mimic (30 nM) or miR-5096-inhibitor (30 nM), were loaded with calcein and labelled with DiL-C18. Calcein diffuses through gap junctions, while DiL-C18 does not. These cells were then plated on unlabeled HMEC monolayer (acceptor). After 5 h of co-culture, HMEC establishing GJIC with U87 become fluorescent by calcein diffusion. Carbenoxolone (100 μM) prevented it without affecting the cell-to-cell adhesion. Dotted areas are enlarged in the right inserts (representative of 3 experiments; Bar 80 μm). **B.** Histogram shows the cell number of HMEC receiving dye (calcein) per U87 (mean ±SD; **P<0.01 *vs* control, *n* = 3). Carb.: 100 μM carbenoxolone. **C.** Immunoblot analysis of Cx43 protein in HMEC (left) and U87 (right) whole cell lysates after 12 h of co-culture. P0, P1 and P2 denote the three major Cx43 migration bands. One representative of 3 independent experiments is shown (β-actin as loading control). **D.** Histogram shows changes in all band intensity related to the total Cx43 expression level in the whole cell lysates (mean ± SD; *P<0.05 *vs* empty; Mann-Whitney U test and Kruskal-Wallis test; *n* = 3).

Transfected U87 were double loaded with calcein, a dye that passes through gap junctions, and DiL, a membrane-bound dye (Figure 3A; [19]). Labelled U87 were plated onto HMEC monolayers to which they rapidly adhered. The calcein transfer to HMEC was then measured, attesting the formation of heterocellular GJIC. When U87 were transfected with a miR-5096 mimic, calcein transfer was significantly increased within 5 hours. The gap junction blocker, carbenoxolone, did not affect cancer cell adhesion to endothelial cells (Figure 3A), but abolished calcein transfer from U87 to HMEC (Figure 3B). A similar result was obtained by inhibiting miR-5096 expression in glioblastoma cells. These results suggest that miR-5096 itself favors the communication between cancer and endothelial cells.

We analyzed the effect of miR-5096 on Cx43 expression in the two cell types (Figure 3C). Transfection of U87 with a miR-5096 mimic induced a 2-fold increase in Cx43 expression in U87, without modifying Cx43 expression in co-cultured HMEC for 12 h (Figure 3D). This is in agreement with the large transfer of miR-5096 from U87 to HMEC within 12 hours of co-culture (Figure 2C, 2D) and its inhibition by decreasing Cx43 expression using siRNA in U87 (see supplementary Figure S3C).

### miR-5096 increases the proangiogenic effect of glioblastoma cells *in vitro*

**Figure 4:**
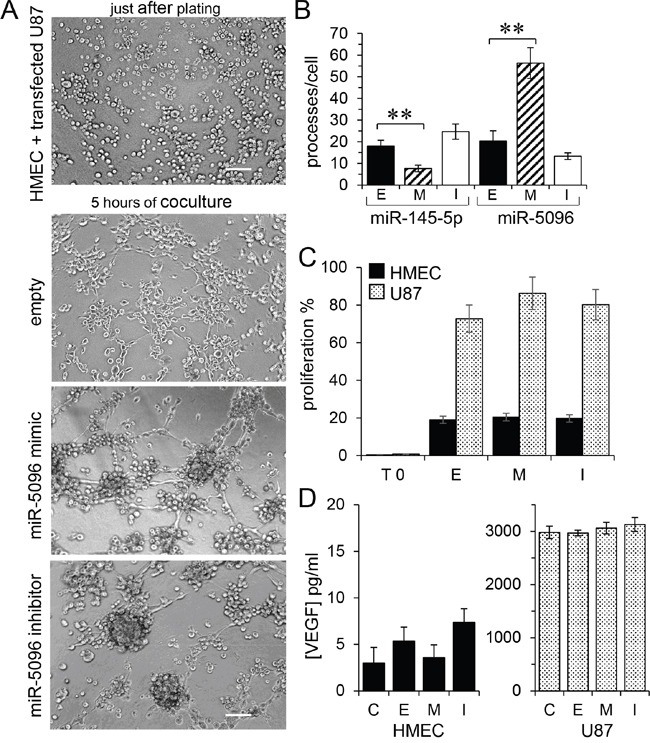
Proangiogenic effect of miR-5096 transfer **A.** *In vitro* tubulogenesis assay of HMEC plated on Matrigel with donor U87, loaded or not (empty) with miR-5096 mimic (30 nM) or inhibitor (30 nM), just after plating (upper panel) or after 5 h of co-culture. Representative micro-photographs of endothelial tube formation (Bar 80 μm; n=3 experiments triplicate). **B.** Comparative effects of miR-145 and miR-5096. Histogram shows the number of branch points per field of view (at least 80 single cells were scored). Donor HMEC were loaded or not (empty; E) with miR-145 mimic (M; 30 nM) or inhibitor (I; 30 nM), and co-cultured with U87 for 5 h (left). Acceptor HMEC were co-cultured with donor U87 as described in panel A (mean ± SD; **P<0.01 *vs* empty; Mann-Whitney U test and Kruskal-Wallis test; *n* = 3). **C.** miR-5096 did not affect the proliferation of HMEC (black) or U87 (dashed), when loaded or not (empty) with miR-5096 mimic (30 nM) or inhibitor (30 nM), and plated separately for 24 h in homotypic culture conditions (T0 initial time point of experiment; mean ± SD; *P*>0.05 *vs* empty; Mann-Whitney U test and Kruskal-Wallis test; *n* = 3). **D.** miR-5096 did not affect the VEGF release by loaded cells. Same experimental conditions in panel C (mean ± SD; *P*>0.05 *vs* C non-transfected cells; Mann-Whitney U test and Kruskal-Wallis test; *n* = 3).

We subsequently used an *in vitro* matrigel tube formation assay to explore if miR-5096 could modulate the ability of HMEC to form capillary-like structures [18]. Co-culture of U87 and HMEC initiated the formation of typical capillary-like structures within 5 hours (Figure 4A). When miR-5096 mimic-loaded U87 were co-cultured with HMEC, the formation of a capillary-like network at 5 hours was increased (Figure 4A, 4B). Such an effect was not observed when U87 were loaded with a miR-5096 specific inhibitor. Thus, miR-5096 expressed by glioblastoma cells could act as a proangiogenic factor, i.e., through increasing tubulogenesis (Figure 4B). The opposite effect was observed when miR-145-5p mimic-transfected HMEC were co-cultured with U87, as this transfection inhibited the formation of capillary-like structures (Figure 4B, see supplementary Figure S1). Altogether, these results suggest that, by modulating the tumor associated capillary-like network, the transfer of miR-145-5p from endothelial to cancer cells may decrease tumor growth whereas the transfer of miR-5096 from cancer to endothelial cells may have opposite effects by promoting angiogenesis.

As previously reported [17], miR-145-5p mimic also decreased U87 cell proliferation (not shown). Conversely, neither cell loading with a miR-5096 mimic nor a miR-5096 inhibitor did affect the proliferation of U87 and HMEC (Figure 4C). The VEGF soluble protein release was not influenced by miR-5096 (Figure 4D).

### miR-5096 disrupts the heterocellular GJIC with time

**Figure 5:**
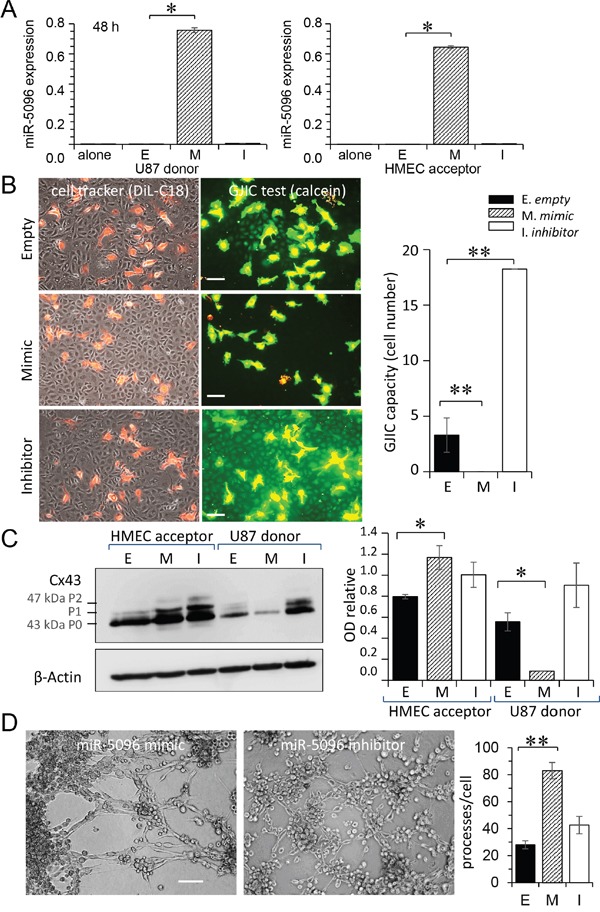
Time-dependent effects of miR-5096 **A.** miR-5096 transfer from U87 to HMEC, two days after loading. Donor U87, either non-transfected (E) or transfected with mimic (M; 30 nM) or inhibitor (I; 30 nM), were cultured alone for 48 h, then co-cultured with acceptor HMEC (right panel) for 12 h. The miR-5096 levels, relative to U6 snRNA, are means ± SD (*P<0.05 *vs* empty; Mann-Whitney U test and Kruskal-Wallis test; *n* = 3). **B.** Blockage of functional GJIC between U87 and HMEC by miR-5096. Transfected U87 were cultured alone for 48 h, then loaded with calcein and DiL-C18. Labelled U87 (donor) were plated onto HMEC monolayer (acceptor) as described in Fig. 3A. Phase-contrast microphotographs after 5 h of co-culture (representative of 3 experiments; Bar 80 μm). Histogram shows the cell number of HMEC receiving dye (calcein) per U87 (mean ±SD, n=3; **P<0.01 *vs* control). **C.** Down-regulation of Cx43 expression in U87 by miR-5096. Immunoblot analysis of Cx43 in whole-cell lysates from transfected U87 (right), cultured alone for 48 h then co-cultured with HMEC for 12h (representative of 3 independent experiments; β-actin as loading control). Histogram shows the miR-5096-mediated down-regulation of Cx43 in U87 and the up-regulation of Cx43 in co-cultured HMEC (mean ± SD; *P<0.05 *vs* empty; Mann-Whitney U test and Kruskal-Wallis test; *n* = 3). **D.** miR-5096 mediated tubulogenesis. Representative micro-photographs of HMEC plated on Matrigel for 5 h with U87 previously loaded with mimic or inhibitor (Bar 80 μm). Histogram showing the number of branch points per field (mean ± SD; **P<0.01 *vs* empty; Kruskal-Wallis test; *n* = 3).

To determine whether miR-5096 effects are stable with time, its cell expression level was determined two days after cell loading. More precisely, donor U87 were plated 2 days after transfection with acceptor HMEC for 12 h (ratio 1:1). As shown in Figure 5A, miR-5096 expression level in U87 was lower after 2 days in culture than within the first day (by 30 %; see Figure 2C). Its expression level in HMEC was also decreased (by 70% of its value measured within the first day; see Figure 2D). Thus, after 2 days, U87 were still capable to transfer miR-5096 to co-cultured HMEC but to a lesser extent.

However, U87 loaded with miR-5096 mimic did not transfer calcein to HMEC monolayers (Figure 5B). Conversely, a large calcein transfer to HMEC, attesting the formation of heterocellular GJIC, was observed by inhibiting miR-5096 in U87. The GJIC capacity was increased by 4 fold with the miR-5096 inhibitor and was completely abolished with miR-5096 mimic (Figure 5B, right panel). We performed the same experiment by transfecting HMEC with miR-5096 mimic and inhibitor. Two days after transfection, loaded HMEC were plated onto HMEC monolayers and the calcein transfer was observed (see Supplementary Figure 2). Neither miR-5096 mimic nor inhibitor affected the GJIC established between transfected and non-transfected HMEC.

Since functional GJIC required Cx43 expression, we performed immunoblot analyses of whole-cell extracts in co-cultures, 2 days after loading (Figure 5C). Strikingly, miR-5096 mimic down-regulated Cx43 expression in transfected U87, while it up-regulated Cx43 expression in HMEC. The immunofluorescent labelling of Cx43 in U87 further demonstrated the time-dependent decrease in heterocellular GJIC (see supplementary Figure S4). These results suggest that miR-5096 modulates Cx43 expression and GJIC, in a time- and cell type-dependent manner. In spite of the decrease in Cx43 expression, U87 loaded with miR-5096 mimic were still able to increase the formation of a capillary-like network at 5 hours (Figure 5D).

## DISCUSSION

Accumulated evidence indicate that non-coding microRNAs (miRs) have multiple effects on gene regulation during tumor progression [14, 21]. Here, we demonstrate the ability of gap junctions to drive miRs exchange between HMEC and the U87 human glioblastoma cell line and to modulate the behavior of target cells. First, miR-145, which is downregulated in early stages of glioma progression [22] and behaves as a tumor suppressor [17], can migrate from endothelial to tumor cells and function as an “antiangiogenic” signal. Second, miR-5096, which is upregulated in glioma (as compared to normal brain tissues) and promotes glioma invasion [4], can migrate from tumor cells to endothelial cells in which it functions as a “proangiogenic” signal. The cell-to-cell transfer of these miRs is inhibited by the loss of contact between HMEC and U87 cells and by the presence of the GJIC blocker, carbenoxolone, indicating that miR-145 and miR-5096 use the same intercellular transfer pathway (GJIC) to mediate their opposite effects on angiogenesis.

The observation that miR-145 can be transferred from HMECs to U87 enforces our previous demonstration that functional gap junctions between colon carcinoma cells and HMEC could permit the transfer of miR-145-5p from cell to cell [18]. Re-expression of miR-145 in U87 can inhibit glioma cells proliferation, invasion and angiogenesis *in vitro* and reduce glioma growth *in vivo* [17]. This effect of miR-145 was initially related to its ability to decrease vascular endothelial growth factor (VEGF) expression levels [17]. Actually, VEGF expression level also decreases when miR-145 expression is downregulated [17]. Moreover, perivascular invasion by glioma cells increases when VEGF synthesis is deficient [23] and when brain tumor xenografts are treated with anti-VEGF blocking antibodies [24, 25]. In patients GBMs that are resistant to bevacizumab therapy, demonstrated a tendency toward perivascular invasion [26]. Blocking angiogenic signaling using antibodies targeting the VEGF-A axis failed to curb progressive tumor growth and meaningfully extend patient survival, suggesting that both perivascular brain tumor growth and invasion use a VEGF-independent mechanism of tumor vascularization [3]. Conversely, we here demonstrate an angiogenic effect of miR-5096, which is independent of any change in VEGF release from U87 cells. Also, overexpression and downregulation of miR-5096 did not affect the proliferation of either U87 or HMEC *in vitro*. Altogether, the transfer of miR via functional gap junctions between tumor cells and endothelial cells may modulate the behavior of tumor cells and surrounding normal tissues and subsequently change tumor growth characteristics [4, 6, 14].

We also demonstrate that miR-5096 transfer via gap junctions is time-dependent. Both the GJIC and underlying Cx43 expression were transiently increased in transfected U87. After a few days in culture, gap junctions between U87 and HMEC have lost their functionality but miR-5096 is still transferred from U87 to HMEC, although less efficiently, suggesting that another still unidentified pathway is activated [27, 28]. Conversely, transfection of miR-5096 into HMEC does not affect the homocellular GJIC in HMEC monolayers (see Supplementary Figure S2), indicating that overexpression of miR differentially regulates Cx43 expression in HMEC and U87 [14, 29, 30].

Altogether, our results highlight a complex dialog between brain blood vessels and perivascular glioma cells that ends in invasion in a VEGF-independent manner. Future investigation will indicate if the manipulation of miR exchanges between these cells deserves to be developed as an alternative anti-GBM therapy.

## MATERIALS AND METHODS

### Cells

Human microvascular endothelial cells (HMEC; Lonza, Switzerland) and glioblastoma cells (U87-MG; ATCC HTB-14) were grown in DMEM plus 10% FCS (5% CO_2_; 37°C). Cells were incubated overnight in FCS-free media before use.

### Reagents

Mouse monoclonal anti-Cx43 (610062) was purchased from BD Transduction Laboratories (Lexington, KY). Mouse anti-Hsc70 and anti-β-actin were from Santa Cruz Biotech. Vybrant cell labeling solution DiL-C18 was from Molecular Probes (Invitrogen; Life Technologies, Saint-Aubin, Fr). Rabbit polyclonal anti-HIF-1α (ab2185) was from Abcam (Paris, Fr). GW4869 was purchased from Calbiochem (Merck Chimie SAS, Fontenay-sous-Bois, Fr). Other chemicals were from Sigma-Aldrich.

### Transfection

Human hsa-miR-145-5p mimics (mirVana TM miRNA mimic, 4464066-MC11480), hsa-miR-145-5p inhibitors (mirVana TM miRNA mimic, 4464084-MH11480), hsa-miR-5096 mimics (mirVana TM miRNA mimic, 4464066-MC22429) and hsa-miR-5096 inhibitors (mirVana TM miRNA mimic, 4464084-MH22429) were purchased from Ambion (Invitrogen). Silencing RNA (siRNA) targeting the human Cx43 gene was purchased from Santa Cruz Biotech (GJA1_human mapping 6q22.31; Clinisciences; Nanterre, Fr) and control siRNA was from Dharmacon (ThermoFischer, Saint-Remy-les-Chevreuses, Fr). Cells were transfected by lipofectamine RNAiMAX according to the manufacturer's protocol (Invitrogen; Life Technologies).

### Co-culture and cell sorting by flow cytometry

Acceptor cells were labelled with DiL-C18, then washed and mixed with unlabeled cells (donors) in a ratio of 1:1. After co-culture, donors and acceptors were separated by flow cytometry based on the fluorescence dye. Cell sorts were carried out twice to guarantee 100% purity.

### RNA isolation and real-time PCR analysis

Total RNA was isolated using TRIzol reagent (Invitrogen). Expression of miR-145 was determined using TaqMan miRNA assay (Invitrogen) according the manufacturer's protocols. Level of miR-145 was expressed relative to the level of U6 snRNA (Ambion, 4427975-001973), used as internal control for each measurement. Relative values thus obtained were averaged.

### Heterocellular GJIC functionality

CRC cells were labeled with 4 μM calcein/AM (30 min) together with 10 μM DiL-C18 as previously detailed [19]. After washing, 10^3^ fluorescent cells were laid on HMEC monolayers. The transfer of dye was visualized after a given time at 37°C.

### Immunoblotting

Briefly, cells were lysed in RIPA buffer, and Western blots were performed with antibodies, as previously described [19].

### Endothelial tube formation assay in collagen gels

Cells were trypsinized and resuspended in ECM gel with DMEM according to the manufacturer's instructions (Cell Biolabs, Inc) [19]. Each well is duplicated for each experiment, and each experiment was repeated three times. For short term assays (after 4 hours of incubation at 37°C), 80 single HMEC cells were scored for the number of processes per cell. Cells were photographed at a magnification of x10 using Zeiss microscope, equipped with a video camera.

### Cell proliferation

CellTrace^™^ Violet was used for tracking proliferation in the two cell types, by fluorescent dye dilution and flow cytometry, according to the manufacturer's protocol (MolecularProbes, ThermoFisher, Fr).

### Human VEGF immunoassay

For collection of conditioned media (CM), cells were grown 6 h in FCS-free DMEM then fresh medium (3 ml/T-25 flask) was added for 12 h before to be collected. The quantitative determinations of human VEGF concentrations in CM were made by enzyme-linked immunosorbent assays (ELISAs; Quantikine; R&D Systems), according to the manufacturer's instructions.

### Statistical analysis

Results are expressed as mean ± SD. Groups were compared using one-way analysis of variance (ANOVA; Statview Software). A Mann-Whitney *U* test was also used to compare data groups. In some cases, statistics were made with Tanagra software using a Kruskal-Wallis 1-way ANOVA. In all cases, *P values < 0.05 were significant.

## SUPPLEMENTARY MATERIALS FIGURES



## References

[1] Dolecek TA, Propp JM, Stroup NE, Kruchko C (2012). CBTRUS statistical report: primary brain and central nervous system tumors diagnosed in the United States in 2005-2009. Neuro Oncol.

[2] Grossman SA, Ye X, Piantadosi S, Desideri S, Nabors LB, Rosenfeld M, Fisher J, Consortium NC (2010). Survival of patients with newly diagnosed glioblastoma treated with radiation and temozolomide in research studies in the United States. Clinical cancer research.

[3] Baker GJ, Yadav VN, Motsch S, Koschmann C, Calinescu AA, Mineharu Y, Camelo-Piragua SI, Orringer D, Bannykh S, Nichols WS, deCarvalho AC, Mikkelsen T, Castro MG, Lowenstein PR (2014). Mechanisms of glioma formation: iterative perivascular glioma growth and invasion leads to tumor progression, VEGF-independent vascularization, and resistance to antiangiogenic therapy. Neoplasia.

[4] Hong X, Sin WC, Harris AL, Naus CC (2015). Gap junctions modulate glioma invasion by direct transfer of microRNA. Oncotarget.

[5] Oliveira R, Christov C, Guillamo JS, de Bouard S, Palfi S, Venance L, Tardy M, Peschanski M (2005). Contribution of gap junctional communication between tumor cells and astroglia to the invasion of the brain parenchyma by human glioblastomas. BMC Cell Biol.

[6] Sin WC, Aftab Q, Bechberger JF, Leung JH, Chen H, Naus CC (2016). Astrocytes promote glioma invasion via the gap junction protein connexin43. Oncogene.

[7] Zhang W, DeMattia JA, Song H, Couldwell WT (2003). Communication between malignant glioma cells and vascular endothelial cells through gap junctions. J Neurosurg.

[8] Kolar K, Freitas-Andrade M, Bechberger JF, Krishnan H, Goldberg GS, Naus CC, Sin WC (2015). Podoplanin: a marker for reactive gliosis in gliomas and brain injury. J Neuropathol Exp Neurol.

[9] Sin WC, Crespin S, Mesnil M (2012). Opposing roles of connexin43 in glioma progression. Biochimica et biophysica acta.

[10] Naus CC, Laird DW (2010). Implications and challenges of connexin connections to cancer. Nature reviews Cancer.

[11] Katakowski M, Buller B, Wang X, Rogers T, Chopp M (2010). Functional microRNA is transferred between glioma cells. Cancer research.

[12] Ambros V (2004). The functions of animal microRNAs. Nature.

[13] Bartel DP (2009). MicroRNAs: target recognition and regulatory functions. Cell.

[14] Shea A, Harish V, Afzal Z, Chijioke J, Kedir H, Dusmatova S, Roy A, Ramalinga M, Harris B, Blancato J, Verma M, Kumar D (2016). MicroRNAs in glioblastoma multiforme pathogenesis and therapeutics. Cancer Med.

[15] Brink PR, Valiunas V, Gordon C, Rosen MR, Cohen IS (2012). Can gap junctions deliver?. Biochimica et biophysica acta.

[16] Valiunas V, Polosina YY, Miller H, Potapova IA, Valiuniene L, Doronin S, Mathias RT, Robinson RB, Rosen MR, Cohen IS, Brink PR (2005). Connexin-specific cell-to-cell transfer of short interfering RNA by gap junctions. The Journal of physiology.

[17] Lu Y, Chopp M, Zheng X, Katakowski M, Wang D, Fraser E, Nguyen M, Jiang F (2015). Overexpression of miR145 in U87 cells reduces glioma cell malignant phenotype and promotes survival after *in vivo* implantation. Int J Oncol.

[18] Thuringer D, Jego G, Berthenet K, Hammann A, Solary E, Garrido C (2016). Gap junction-mediated transfer of miR-145-5p from microvascular endothelial cells to colon cancer cells inhibits angiogenesis. Oncotarget.

[19] Thuringer D, Berthenet K, Cronier L, Jego G, Solary E, Garrido C (2015). Oncogenic extracellular HSP70 disrupts the gap-junctional coupling between capillary cells. Oncotarget.

[20] Thuringer D, Berthenet K, Cronier L, Solary E, Garrido C (2015). Primary tumor- and metastasis-derived colon cancer cells differently modulate connexin expression and function in human capillary endothelial cells. Oncotarget.

[21] Ling H, Fabbri M, Calin GA (2013). MicroRNAs and other non-coding RNAs as targets for anticancer drug development. Nat Rev Drug Discov.

[22] Lu Y, Chopp M, Zheng X, Katakowski M, Buller B, Jiang F (2013). MiR-145 reduces ADAM17 expression and inhibits *in vitro* migration and invasion of glioma cells. Oncology reports.

[23] Blouw B, Song H, Tihan T, Bosze J, Ferrara N, Gerber HP, Johnson RS, Bergers G (2003). The hypoxic response of tumors is dependent on their microenvironment. Cancer Cell.

[24] Rubenstein JL, Kim J, Ozawa T, Zhang M, Westphal M, Deen DF, Shuman MA (2000). Anti-VEGF antibody treatment of glioblastoma prolongs survival but results in increased vascular cooption. Neoplasia.

[25] de Groot JF, Fuller G, Kumar AJ, Piao Y, Eterovic K, Ji Y, Conrad CA (2010). Tumor invasion after treatment of glioblastoma with bevacizumab: radiographic and pathologic correlation in humans and mice. Neuro Oncol.

[26] Clark AJ, Lamborn KR, Butowski NA, Chang SM, Prados MD, Clarke JL, McDermott MW, Parsa AT, Berger MS, Aghi MK (2012). Neurosurgical management and prognosis of patients with glioblastoma that progresses during bevacizumab treatment. Neurosurgery.

[27] Chen X, Liang H, Zhang J, Zen K, Zhang CY (2012). Secreted microRNAs: a new form of intercellular communication. Trends Cell Biol.

[28] Salido-Guadarrama I, Romero-Cordoba S, Peralta-Zaragoza O, Hidalgo-Miranda A, Rodriguez-Dorantes M (2014). MicroRNAs transported by exosomes in body fluids as mediators of intercellular communication in cancer. Onco Targets Ther.

[29] Ivashchenko A, Berillo O, Pyrkova A, Niyazova R, Atambayeva S (2014). The properties of binding sites of miR-619-5p, miR-5095, miR-5096, and miR-5585-3p in the mRNAs of human genes. BioMed research international.

[30] Vinken M (2016). Regulation of connexin signaling by the epigenetic machinery. Biochimica et biophysica acta.

