# Supplementary material for: Transfer of functional microRNAs between glioblastoma and microvascular endothelial cells through gap junctions
Source: Oncotarget. 2016 Sep 20;7(45):73925–34. doi: 10.18632/oncotarget.12136 (PMC5342024; doi:10.18632/oncotarget.12136)
Supplement: Supplementary file 1 [file oncotarget-07-73925-s001.pdf]

# Transfer of functional microRNAs between glioblastoma and microvascular endothelial cells through gap junctions

## SUPPLEMENTARY FIGURES

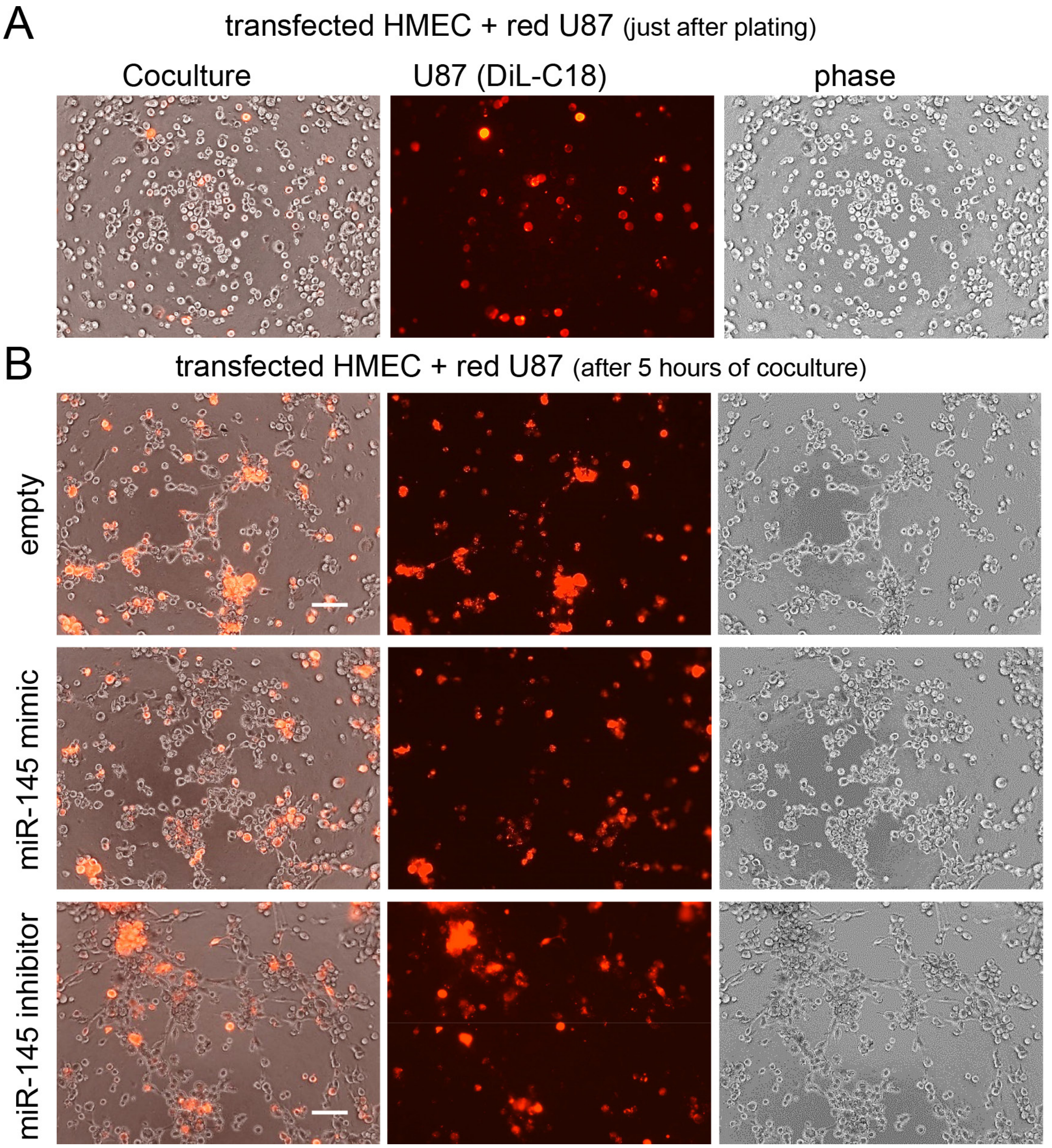


**Supplementary Figure S1: Antiangiogenic effect of miR-145 transfer.** *In vitro* tubulogenesis assay of HMEC loaded or not (empty) with miR-145-5p mimic (30 nM) or inhibitor (30 nM). Donor HMEC were plated with red labelled U87 on Matrigel, and incubated for 5 h. **A.** Micro-photographs of cells just after plating. **B.** Representative micro-photographs of endothelial tube formation (Bar 80 μm; n=3 experiments triplicate).

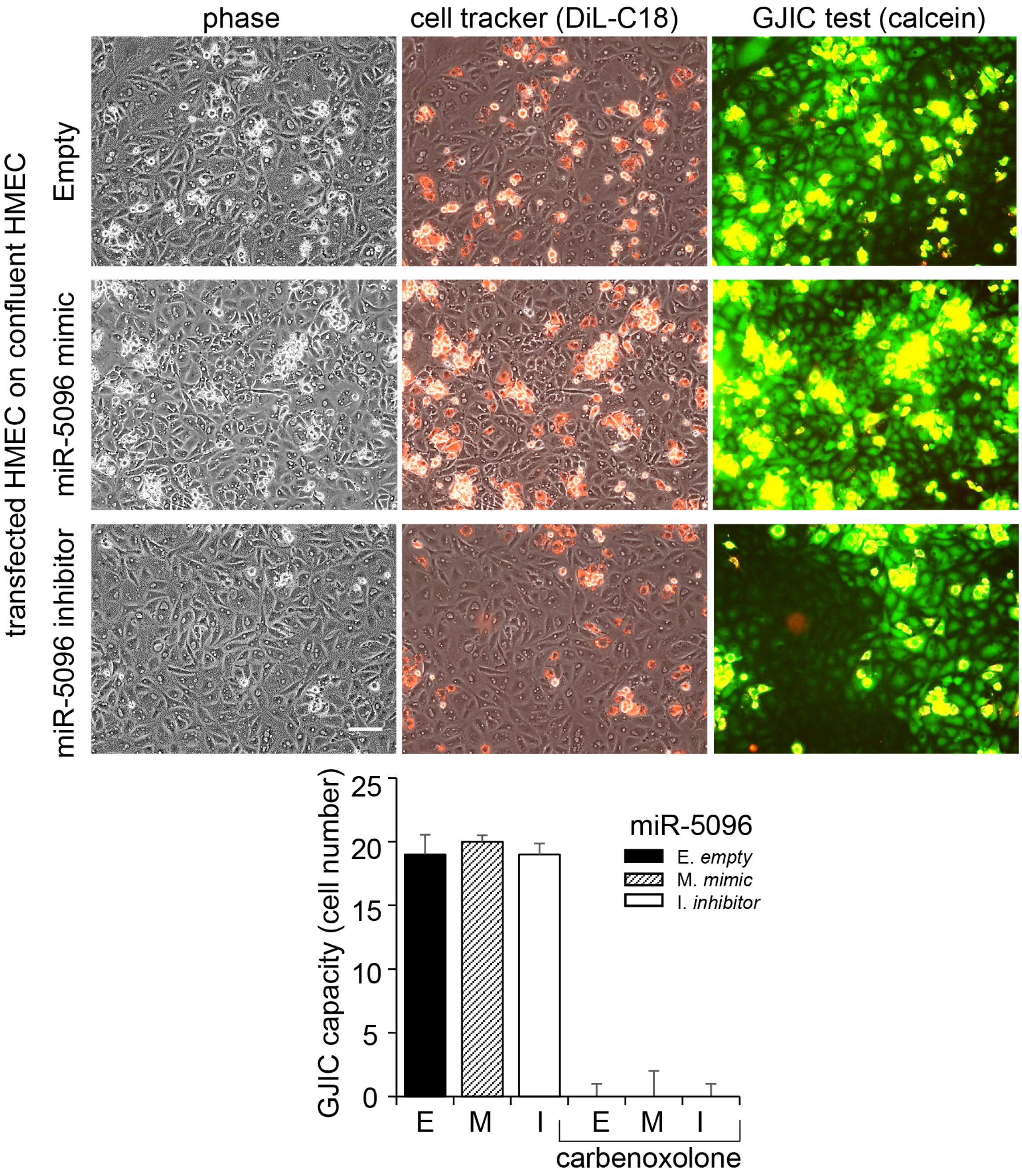


**Supplementary Figure S2: miR-5096 does not affect the GJIC in HMEC monolayers.** Donor HMEC, preloaded or not (E) with miR-5096-mimic (30 nM) or miR-5096-inhibitor (30 nM), were cultured alone for 48h, then loaded with calcein/AM and DiL-C18. Labelled HMEC (donor) were plated onto unlabeled HMEC monolayer (acceptor). HMEC establishing GJIC with labelled transfected HMEC became fluorescent by calcein diffusion. Microphotographs after 5 h of coculture ($n$ = 5; Bar 80 µm). Histogram shows the cell number of HMEC receiving dye (calcein) per donor HMEC. Carbenoxolone (100 µM) improved it (mean ±SD, n=3).

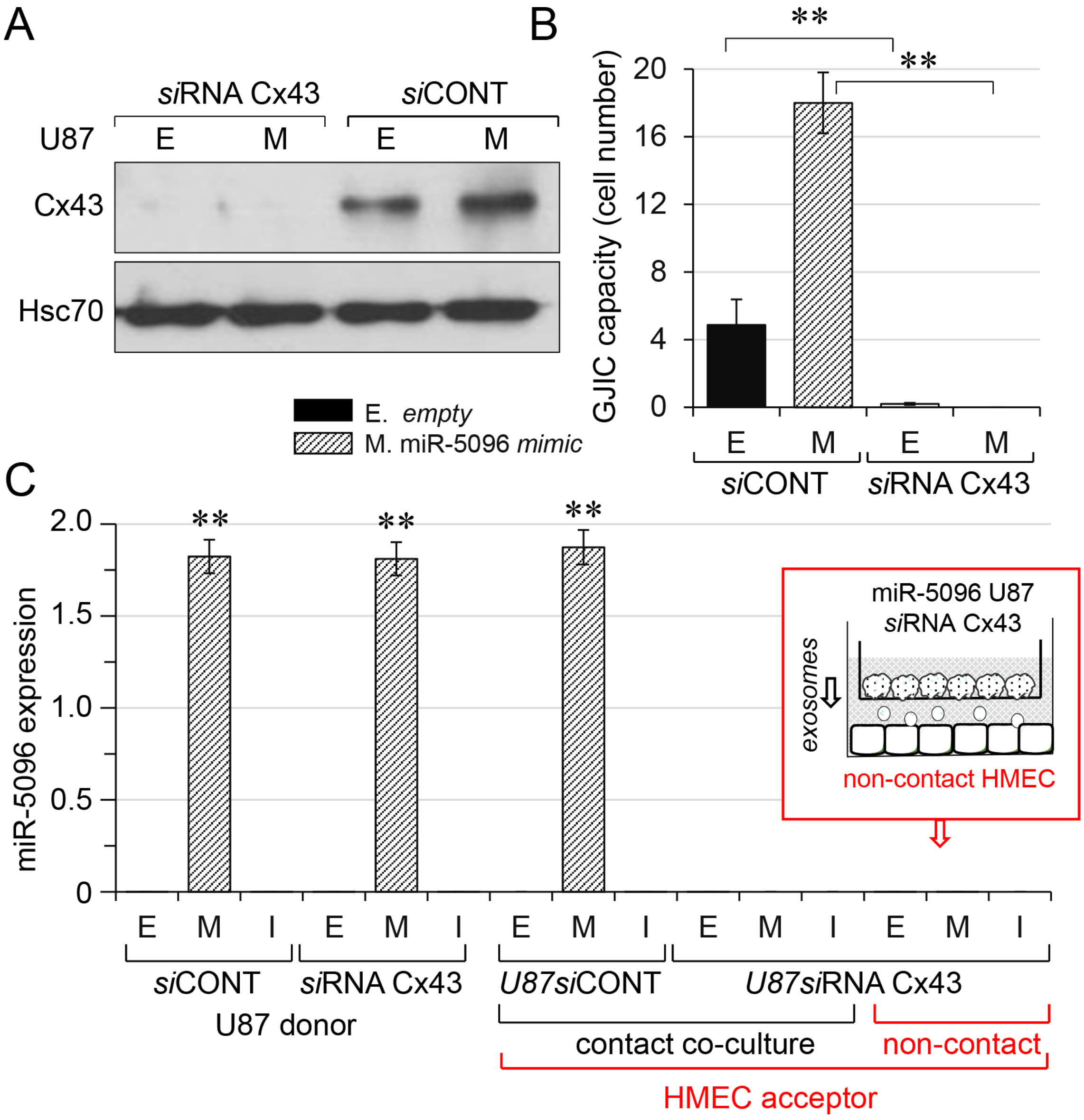


**Supplementary Figure S3: Contribution of Cx43 to the miR-5096 transfer from U87 to HMEC within 12 h of co-culture. A.** Western blot showing silencing RNA mediated knockdown of Cx43 expression in U87, loaded (M) or not (E) with miR-5096 mimic. *si*RNA Cx43 (GJA1_human mapping 6q22.31); *si*CONT: control *si*RNA (n=3; Hsc70 as loading control). **B.** *si*RNA mediated knockdown of Cx43 expression in U87 suppresses gap junction dye coupling to HMEC. Histogram shows the cell number of HMEC receiving dye (calcein) per U87 (mean ±SD; $^{**}P<0.01$ *vs* control *si*RNA, $n = 3$). **C.** Down-regulation of Cx43 expression in U87 does not affect loading of miR-5096 mimic but suppresses transfer of miR-5096 to HMEC. Right insert: scheme illustrating the procedure to evaluate the contribution of paracrine pathways to the intercellular miR transfer. Acceptor HMEC were co-cultured with *si*RNA-transfected U87 with no physical contact (non-contact). Donor U87 were loaded or not (E) with miR-mimic (M; 30 nM) or miR-inhibitor (I; 30 nM). The miR levels were determined by qPCR in donors (left) and acceptors (right), after 12 h of co-culture, and measured relative to U6 snRNA. Values are means ± SD of triplicate measurements from three experiments; $^{**}P<0.01$ *vs* empty (Mann-Whitney U test and Kruskal-Wallis test; $n = 3$).

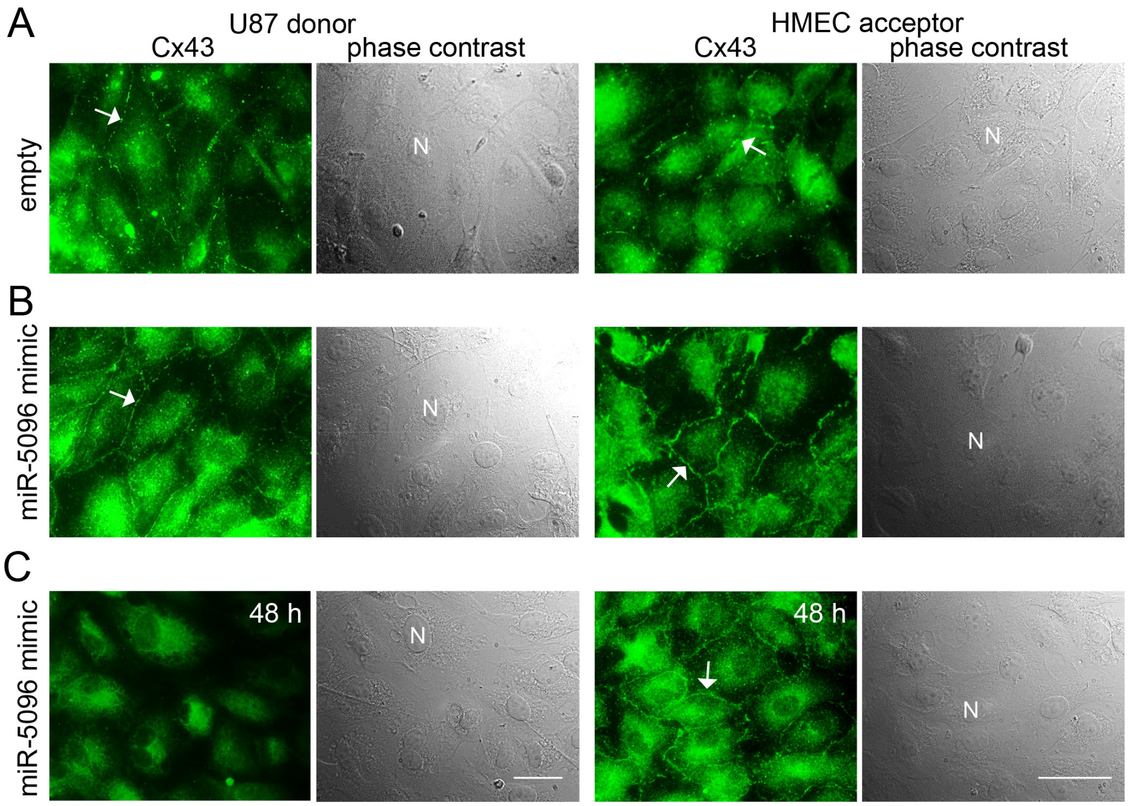


**Supplementary Figure S4: Immunolocalization of Cx43. Donor U87, loaded (B, C) or not (A, empty) with miR-5096 mimic (30 nM), were plated with HMEC and incubated for 12 h (A, B) and 48 h (C).** Cultured cells were permeabilized with Triton X100 (0.2%) and immunolabelled with monoclonal anti-Cx43 antibody (610061) followed by goat anti-mouse antibody Alexa 488 nm. Polyclonal anti-Von willebrand factor (A0082, Dako) was used to identify endothelial cell clusters. **A.** In control conditions (empty), a punctate labelling was observed at sites of cell-cell apposition (*arrows*). N, *nucleus*. **B.** In mimic transfected conditions and after 12 h of co-culture, almost continuous gap junctions plaques were seen at the cell-cell contacts in U87 and in HMEC. **C.** In mimic transfected conditions and after 48h of co-culture, the plasma membrane staining with Cx43 persisted in HMEC but disappeared in U87. Representative micro-photographs of endothelial and glioblastoma cell clusters (Bar 60 µm; n=2 experiments).
